# Gas Stoves, &c.

**Published:** 1893-08-26

**Authors:** 


### PRACTICAL DEPARTMENTS.

### GAS STOVES, &c.

We have frequently advocated the more extensive use of gas for cooking purposes, and the sight of a specially attractive catalogue from Messrs. James Slater and Co., of High Holborn, impels again a few words on this subject. The illustrations before us are chiefly of stoves and coppers on a large scale. Hot closets and heating apparatus of all kinds are to be seen at Messrs. Slaters', and a very good general idea of them can be obtained from a careful study of these drawings, printed on separate sheets and enclosed in a particularly neat portfolio.

There can be no doubt that gas as a cooking agent will be increasingly used every year. Its superior claims on the score of cleanliness, and the easier regulation of heat, to say nothing of economy, are sure to advance its popularity, and as its management is better and better understood there will be less chance of objections being raised on account of possible dangers or disagreeables from smell or otherwise.

Where it is not feasible for the whole of the cooking to be done by this means, a small apparatus fixed at the side of the ordinary kitchen range will be found of great use.

We recommend all who are considering the advisability of adopting gas in their kitchens, either wholly or in part, to apply to Messrs. Slater for their illustrated descriptions of "warming, lighting, and cooking" appurtenances.

